# Advancing Heart Failure Care: Breakthroughs and Emerging Strategies

**DOI:** 10.3390/jcm14207253

**Published:** 2025-10-14

**Authors:** Andrea Tedeschi, Federico Barocelli, Luigi Gerra, Federico Breviario, Matteo Palazzini, Nicolina Conti, Stefano Ferraro, Maria Giulia Bolognesi, Francesco Di Spigno, Piero Gentile, Andrea Garascia, Enrico Ammirati, Giulia Magnani, Giampaolo Niccoli, Nuccia Morici, Daniela Aschieri

**Affiliations:** 1 Cardiology Unit, Guglielmo da Saliceto Hospital, 29121 Piacenza, Italy; l.gerra@ausl.pc.it (L.G.); f.breviario@ausl.pc.it (F.B.); s.ferraro@ausl.pc.it (S.F.); m.bolognesi@ausl.pc.it (M.G.B.); francesco.dispigno@yahoo.com (F.D.S.); d.aschieri@ausl.pc.it (D.A.); 2 Cardiology Division, Parma University Hospital, 43126 Parma, Italy; federico.barocelli@gmail.com (F.B.); giuliamagnani.cardio@gmail.com (G.M.); giampaolo.niccoli@unipr.it (G.N.); 3 De Gasperis” Cardio Center, Niguarda Hospital, ASST Grande Ospedale Metropolitano Niguarda, 20158 Milan, Italy; matteo.palazzini@ospedaleniguarda.it (M.P.); nicolina.conti@ospedaleniguarda.it (N.C.); piero.gentile@ospedaleniguarda.it (P.G.); andrea.garascia@ospedaleniguarda.it (A.G.); enrico.ammirati@ospedaleniguarda.it (E.A.); 4 IRCCS Fondazione Don Carlo Gnocchi, 20148 Milan, Italy; nmorici@dongnocchi.it

**Keywords:** heart failure, progress, uptitration of GDMT, finerenone, GLP-1 receptor agonists, donation after circulatory death, cell-free DNA, tricuspid regurgitation, total artificial heart

## Abstract

Heart failure represents a complex clinical syndrome characterized by progressive ventricular dysfunction, systemic congestion, and high mortality despite significant advances in pharmacological and device-based therapy. This review explores recent developments across the heart failure continuum, with a focus on therapeutic advances across the continuum of care, with emphasis on both established and emerging strategies. In patients with reduced ejection fraction, early initiation of the four pillars markedly lowers cardiovascular events, yet real-world implementation remains limited by therapeutic inertia and underdosing. Novel agents such as finerenone provide cardiorenal benefits in patients with diabetes and chronic kidney disease, while glucagon-like peptide-1 receptor agonists show promise in preserved or mildly reduced ejection fraction, particularly with obesity. Tricuspid regurgitation, once considered a secondary phenomenon, is now recognized as a modifiable contributor to disease progression, with transcatheter interventions offering new therapeutic avenues. In advanced disease, innovations including donation after circulatory death and the development of total artificial heart systems offer promising solutions to overcome organ shortages and improve access to transplantation. Together, these advances highlight a shift toward precision-guided, multidisciplinary heart failure care.

## 1. Introduction

**Figure 1:**
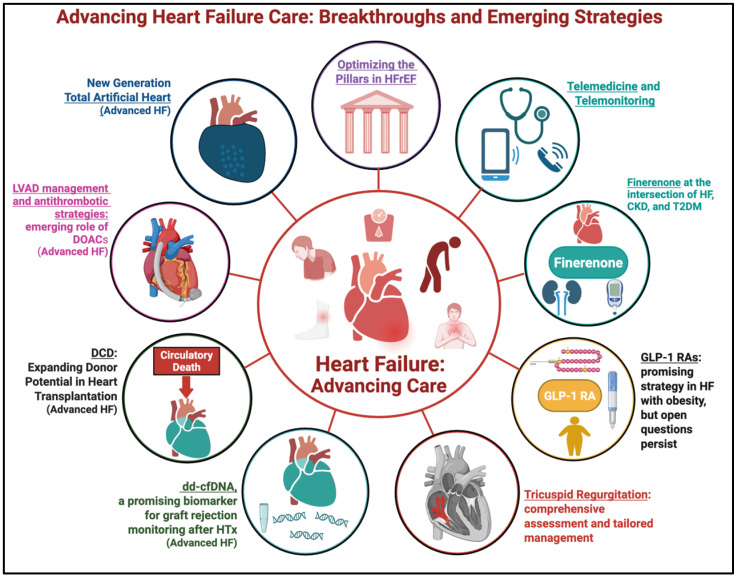
Advancing Heart Failure Care: Breakthroughs and Emerging Strategies. Abbreviations: CKD = Chronic Kidney Disease; DCD = Donation after Circulatory Death; dd-cfDNA = donor-derived cell-free Deoxyribonucleic Acid; DOACs = Direct Oral Anticoagulants; GLP-1 RA = Glucagon-Like Peptide-1 receptor agonist; HF = Heart Failure; HFrEF = Heart Failure with Reduced Ejection Fraction; HTx =Heart Transplantation; LVAD = Left Ventricular Assist Device; T2DM = type 2 diabetes mellitus. This figure was created with BioRender.com (accessed on 28 August 2025).

Heart failure (HF) remains a major global health challenge, driving substantial morbidity, mortality, and healthcare costs despite significant therapeutic advances over the past two decades [1]. It represents a dynamic and heterogeneous syndrome—ranging from early-stage subclinical abnormalities to end-stage disease with refractory symptoms and multi-organ dysfunction [2]. Within this spectrum, heart failure with reduced ejection fraction (HFrEF) has undergone a therapeutic transformation with the advent of four cornerstone drug classes: angiotensin receptor–neprilysin inhibitors (ARNIs), or alternatively angiotensin-converting enzyme inhibitors (ACEis) or angiotensin receptor blockers (ARBs); beta-blockers (BBs); mineralocorticoid receptor antagonists (MRAs); and sodium–glucose cotransporter 2 inhibitors (SGLT2is) [3,4]. These agents have significantly reduced mortality and hospitalization risk. However, real-world translation of clinical trial efficacy remains suboptimal due to delays in initiation, underuse of combination therapy, and failure to achieve target doses [5]. Emerging strategies—such as structured titration protocols, remote monitoring, and telemedicine—are beginning to bridge this gap and redefine models of care [6]. Meanwhile, novel pharmacological targets and device-based strategies are rapidly expanding the HF therapeutic arsenal. Agents like finerenone and Glucagon-like peptide-1 receptor agonists (GLP-1 RAs) are gaining traction for their cardio–renal–metabolic benefits [7,8], while transcatheter therapies for tricuspid regurgitation (TR) are shifting TR from a neglected finding to an actionable contributor to disease progression [9]. In advanced HF, innovations in both transplantation and mechanical circulatory support (MCS) are opening new frontiers. Donation after circulatory death (DCD) offers a promising solution to donor shortages, while donor-derived cell-free deoxyribonucleic acid (dd-cfDNA) is emerging as a non-invasive biomarker for allograft rejection [10,11]. The evolving role of direct oral anticoagulants (DOACs) in left ventricular assist device (LVAD) patients and the development of next-generation total artificial hearts, such as the CARMAT device, highlight the continued trajectory toward individualized and technologically integrated care [12,13]. This review synthesizes the latest evidence across the HF spectrum—from foundational pharmacotherapy to cutting-edge interventions—with a focus on clinical implementation, therapy optimization, and emerging tools that may shape the next era of HF management (Figure 1). Unlike prior reviews, our aim is to critically analyze the data supporting those therapies and strategies that most convincingly promise to improve prognosis across the heterogeneous spectrum of HF, thereby offering clinicians a focused synthesis of the areas with greatest potential clinical impact.

## 2. Optimizing the Four Pillars in Heart Failure with Reduced Ejection Fraction: Uptitration, Gaps, and System-Level Strategies

Contemporary management of HFrEF is anchored in four pharmacologic pillars—ARNIs or, alternatively, ACEis or ARBs; BBs; MRAs; and SGLT2is. These agents have consistently reduced cardiovascular (CV) mortality and hospitalizations, yet their potential is often underexploited in routine practice because of therapeutic inertia, delayed initiation, and suboptimal dosing. A more systematic examination of recent evidence highlights both the magnitude of these gaps and emerging strategies to overcome them. Evidence for early and aggressive titration has been provided by the recent Safety, Tolerability and Efficacy of Rapid Optimization heart failure (STRONG-HF) randomized trial, which demonstrated that rapid initiation and up-titration of oral therapies, initiated before discharge and supported by close follow-up, led to a marked reduction in death and HF rehospitalizations [14]. Despite some limitations—such as selective enrollment and limited SGLT2is use—this study confirmed the safety and benefit of an intensive approach in hemodynamically stable patients [14]. In contrast, real-world registries underscore the persistent gap between trial efficacy and daily practice (Table 1). The multinational EVOLUTION-HF (Utilization of Dapagliflozin and Other Guideline Directed Medical Therapies in Heart Failure Patients: A Multinational Observational Study Based on Secondary Data) study revealed delayed adoption of novel therapies, frequent discontinuation of traditional agents, and only modest persistence with ARNIs and SGLT2is initiation after HF hospitalization [15]. Similarly, data from the GWTG-HF (Get With The Guidelines—Heart Failure) registry in the United States showed that fewer than 20% of eligible patients received an SGLT2is after discharge, even in the presence of type 2 diabetes mellitus (T2DM) or chronic kidney disease (CKD) [16]. These findings highlight the inertia that continues to undermine implementation. At the same time, registry-based initiatives illustrate how structured models of care can improve adoption. In the Netherlands, the prospective nationwide TITRATE-HF registry showed that quadruple therapy was achieved in 44% of patients with chronic or worsening HFrEF, though only 1% reached full target doses, with better results in specialized HF clinics [17]. The Italian BRING-UP-3 initiative further confirmed the advantages of structured outpatient management, with quadruple therapy prescribed in 65% of ambulatory patients, and high uptake of SGLT2is even in HFmrEF and HF with preserved ejection fraction (HFpEF) [18]. These data collectively suggest that system-level approaches, including dedicated HF clinics, are key to improving therapy delivery. Beyond therapeutic inertia, implementation is further limited by practical barriers, including socioeconomic disparities, drug costs, prescription restrictions, and limited access to specialized HF clinics. Patient-related factors such as frailty, polypharmacy, and low health literacy also contribute, highlighting the need for system-level approaches to support long-term adherence. Finally, digital health and telemedicine are emerging as additional tools to accelerate optimization of guideline- direct medical therapy (GDMT) [6]. The Medly Titrate study demonstrated that remote multiparametric monitoring combined with teleconsultations facilitates faster achievement of target GDMT doses without excess adverse events [19]. Similarly, the randomized ADMINISTER (Assessment of Digital Consults in Heart Failure Management regarding Clinical Impact, Safety, and Efficacy Using a Randomized Controlled Trial) trial showed that digital follow-up significantly increased the proportion of patients reaching optimal GDMT within 12 weeks [20]. Finally, a study by Tedeschi et al. observed an overall upward trend in the use of all recommended therapeutic classes during follow-up, with statistical significance reached for increased use of SGLT2is and reduced reliance on diuretics [21]. Collectively, these findings underscore that the success of HFrEF management depends not only on the prescription of evidence-based therapies, but also on structured delivery systems that promote early initiation, titration to target doses, and sustained patient engagement. Bridging the gap between clinical trial efficacy and real-world outcomes remains a cardinal priority in modern HF care.

## 3. Finerenone at the Intersection of Heart Failure, Chronic Kidney Disease, and Type 2 Diabetes Mellitus

Aldosterone and mineralocorticoid receptor (MR) overactivation represent key drivers of inflammation, fibrosis, and adverse remodeling across the heart, kidney, and vasculature. These effects are particularly relevant in patients with comorbid HF, CKD, and T2DM, where renin–angiotensin–aldosterone system (RAAS) dysregulation accelerates disease progression [22,23]. Historically, steroidal MRAs such as spironolactone and eplerenone have been foundational therapies in HFrEF [24]. However, their broader use has been constrained by hyperkalemia, endocrine side effects, and renal limitations [25]. Finerenone (BAY 94-8862) a non-steroidal, third-generation MRA, offers a more favorable pharmacodynamic and safety profile [26]. Unlike steroidal MRAs, which act as partial agonists in cofactor recruitment, finerenone inhibits MR–aldosterone complex activity by blocking the recruitment of transcriptional cofactors such as steroidal coactivator 1 (SRC1) and RNA polymerase II to the regulatory region of the epithelial sodium channel (ENaC) gene [27]. In preclinical studies, finerenone also enhanced nitric oxide bioavailability, reduced reactive oxygen species, and attenuated myocardial fibrosis and inflammation [28]. From a pharmacokinetic perspective, finerenone distributes evenly between the heart and kidneys, in contrast to steroidal MRAs, which preferentially accumulate in renal tissue. This balanced distribution enables parallel anti-fibrotic and anti-inflammatory effects in both organs, translating into slower progression of both HF and CKD. This property, along with its short half-life (2–3 h) and absence of active metabolites, may underlie its lower incidence of hyperkalemia and reduced sexual side effects [29,30]. Its non-steroidal structure minimizes off-target interactions with androgen and progesterone receptors, thereby avoiding gynecomastia, menstrual irregularities, and libido changes commonly seen with spironolactone [31,32]. This improved tolerability facilitates long-term adherence. By selectively blocking MR–aldosterone cofactor recruitment, finerenone attenuates fibrotic remodeling, oxidative stress, and potassium imbalance. These mechanisms may explain the observed reductions in sudden cardiac death and atrial fibrillation in pooled analyses. Compared with spironolactone and eplerenone, finerenone therefore combines a more favorable safety profile with fewer endocrine side effects and lower hyperkalemia risk, although its evidence base in HFrEF remains more limited. Phase III trials have primarily evaluated finerenone in patients with CKD and T2DM, conditions frequently coexisting with HF. Initial evidence from the ARTS (MinerAlocorticoid Receptor Antagonist Tolerability Study) and ARTS-HF (MinerAlocorticoid Receptor antagonist Tolerability Study Heart Failure) studies demonstrated comparable reductions in natriuretic peptides and albuminuria with fewer hyperkalemia events compared to spironolactone or eplerenone in patients with HFrEF and mild-to-moderate CKD and/or T2DM [33,34]. The pivotal FIDELIO-DKD (FInerenone in reducing kiDnEy faiLure and dIsease prOgression in Diabetic Kidney Disease) [35] and FIGARO-DKD (FInerenone in reducinG cArdiovascular moRtality and mOrbidity in Diabetic Kidney Disease) [36] trials, and their pooled analysis FIDELITY, enrolled over 13,000 patients with CKD and T2DM, showing that finerenone significantly reduced major adverse CV events and slowed CKD progression. In FIDELITY, finerenone reduced the risk of new-onset HF (1.9% vs. 2.8%; HR 0.68; 95% CI, 0.50–0.93) and lowered the composite endpoint of CV death or first HF hospitalization (HR 0.82; 95% CI, 0.70–0.95), with a 29% reduction in first HF hospitalization (HR 0.71; 95% CI, 0.56–0.90) [37]. Moreover, pooled data suggested a potential reduction in sudden cardiac death and new-onset atrial fibrillation, possibly linked to improved potassium homeostasis and anti-fibrotic effects [38]. Finally, the FINEARTS-HF (Finerenone in Heart Failure with Mildly Reduced or Preserved Ejection Fraction) trial evaluated finerenone in 6001 patients with symptomatic HF and left ventricular ejection fraction (LVEF) ≥ 40% (mean 53%) [39]. Finerenone significantly reduced the composite endpoint of CV death or total (first and recurrent) HF hospitalizations, with 624 vs. 719 patients experiencing events (relative risk 0.84; 95% CI 0.74–0.95; *p* < 0.05) and an 18% reduction in total HF events (risk ratio 0.82; 95% CI 0.71–0.94). CV mortality was numerically lower (8.1% vs. 8.7%) but not statistically significant. Benefits were consistent across subgroups (with and without T2D or CKD) and the safety profile was favorable, with manageable hyperkalemia and no excess discontinuation or serious adverse events [39]. Collectively, evidence from ARTS, ARTS-HF, FIDELIO-DKD, FIGARO-DKD, and their pooled analysis FIDELITY (Table 2) highlights finerenone as a promising therapeutic option at the intersection of HF, CKD, and T2DM. The 2023 European Society of Cardiology (ESC) HF guideline update recommends the use of finerenone as a Class I indication in patients with T2DM and CKD, underscoring its ability to expand therapeutic opportunities in populations at high risk of hyperkalemia and endocrine side effects [1]. Ongoing studies, such as the CONFIDENCE trial, are exploring its combination with SGLT2is, potentially redefining GDMT for HF [40].

## 4. Exploring the Potential of GLP-1 Receptor Agonists in Heart Failure: Promising Results and Unanswered Questions

Originally developed for the management of T2DM, GLP-1 RAs have revolutionized treatment paradigms in cardiometabolic medicine, demonstrating substantial benefits in weight reduction, glycemic control, and CV outcomes [41,42]. In individuals with HF, particularly those with comorbid obesity or T2DM, GLP-1 RAs have emerged as agents of growing interest, though their precise role remains incompletely defined [8]. GLP-1 is an incretin hormone secreted in response to nutrient intake [43]. Its physiological actions include glucose-dependent insulin secretion, inhibition of glucagon release, delayed gastric emptying, appetite suppression, and enhanced satiety [44]. GLP-1 receptors are expressed not only in pancreatic islets but also in the CV system, including cardiomyocytes, endothelium, and vascular smooth muscle cells. These widespread receptor sites provide a rationale for exploring GLP-1 RAs in CV disease [45,46]. Pharmacologically, GLP-1 RAs are engineered to resist degradation by dipeptidyl peptidase-4 (DPP-4), extending their half-life and enabling sustained receptor activation [47,48]. Preclinical studies in rodent and canine HF models have shown that GLP-1 RAs improve LVEF, reduce myocardial inflammation and apoptosis, enhance glucose uptake in cardiomyocytes, and improve survival by attenuating oxidative stress and TNF signaling [49,50,51,52,53,54]. These findings have prompted clinical trials assessing the CV effects of GLP-1 RAs in HF populations. Notably, the AMPLITUDE-O trial showed that efpeglenatide reduced HF hospitalizations in high-CV risk T2DM patients (HR 0.61; 95% CI 0.38–0.98) [55], while the Harmony Outcomes trial reported similar findings with albiglutide (HR 0.71; 95% CI 0.53–0.94) [56]. A meta-analysis including these and other studies confirmed an overall 11% relative reduction in HF hospitalization risk (HR 0.89; 95% CI 0.82–0.98) [57]. More recently, a pooled analysis focused on patients with HFpEF and HF with mildly reduced EF (HFmrEF) treated with semaglutide or tirzepatide showed a 41% reduction in worsening HF episodes (RR 0.59; 95% CI 0.45–0.76), underscoring their potential in this phenotype [58]. However, clinical outcomes in HFrEF remain inconsistent. The EXSCEL trial, evaluating exenatide in a broad T2DM population, found a signal for harm in patients with LVEF <40%, with increased HF hospitalizations (OR 1.70; 95% CI 1.02–2.83) in this subgroup [59]. Similarly, the LIVE trial, a randomized study of liraglutide in stable HFrEF patients, showed no significant improvements in LVEF after 24 weeks [60]. These discrepancies raise questions about class heterogeneity, differences in pharmacokinetics, or potentially negative inotropic effects in certain settings. Indeed, short-acting agents with higher central nervous system penetrance or variable receptor kinetics may elicit distinct CV responses. Mirroring the trajectory of SGLT2is, GLP-1 RAs are also revealing cardioprotective potential beyond HF, particularly in novel areas such as cardio-oncology [61,62,63]. Key GLP-1 RAs studies in HF are summarized in Table 3. In conclusion, while GLP-1 RAs show promise in reducing HF hospitalizations, their role in improving outcomes for HF patients, particularly those with reduced EF, remains uncertain. Further studies are needed to confirm their efficacy and safety, particularly when combined with GDMT for HFrEF. Despite the growing enthusiasm, the integration of GLP-1 RAs into HF-specific guidelines remains premature. While their benefit in HFpEF or patients with obesity and preserved systolic function appears promising, data in HFrEF are limited, with concerns about safety and uncertain class effects. Most trials of GLP-1 RAs have not tested them on a background of optimized GDMT, leaving uncertainty about additive benefit or interactions. Unlike SGLT2is—firmly established as a GDMT cornerstone across the EF spectrum—GLP-1 RAs are not yet guideline-recommended, though emerging data suggest potential in patients with obesity, T2DM, or preserved LVEF. Their evidence in reduced EF remains limited and conflicting, making ongoing and future trials crucial to clarify their role and possible synergy with SGLT2is and MRAs.

## 5. Tricuspid Regurgitation: From Neglected Lesion to Therapeutic Priority in HF

TR, historically overlooked and deemed secondary to left-sided valvular disease, has emerged as a key contributor to HF progression. Affecting over 4 million people across Europe and the USA, TR is present in up to 50% of HF patients and is independently associated with reduced survival, impaired quality of life (QoL), and organ dysfunction [64]. Right-sided volume overload leads to annular and right ventricular (RV) dilation, initiating a vicious cycle of worsening TR and RV failure. Furthermore, RV enlargement can impair left ventricular (LV) filling through ventricular interdependence, compounding hemodynamic compromise even in patients with preserved LV systolic function [64,65]. Evidence supports TR as an active prognostic factor rather than a passive marker. In a cohort of over 13,000 patients with HFrEF, greater TR severity was directly correlated with 10-year mortality (from 14% in trivial to 86% in severe TR) [65]. Similarly, TR has been identified as an independent predictor of poor outcomes in patients with LV dysfunction and pulmonary hypertension [66]. Etiologically, TR is classified as primary (5–10%), secondary (>90%), or device-related [67]. Primary TR stems from intrinsic leaflet abnormalities, whereas secondary TR, often functional, stems from annular dilation and RV remodeling due to pulmonary hypertension or chronic left-sided HF. Atrial secondary TR (A-STR), increasingly recognized, arises from atrial fibrillation or HF with preserved ejection fraction (HFpEF), leading to annular enlargement without significant RV involvement. Cardiac implantable electronic devices (CIEDs) account for an additional subset of secondary TR [67,68]. Accurate assessment of TR is essential to guide management [69]. Transthoracic and transesophageal echocardiography remain first-line tools, supported by 3D imaging and cardiac magnetic resonance when needed. A comprehensive, multiparametric approach is recommended, incorporating RV dimensions, annular dilation, regurgitation severity, and RV–pulmonary artery (PA) coupling (e.g., tricuspid annular plane systolic excursion/pulmonary artery systolic pressure (TAPSE/PASP) ratio), which strongly predicts clinical outcomes [69,70,71]. Right heart catheterization may help clarify discordant findings [70,71]. Surgical intervention has traditionally been reserved for severe TR in patients undergoing concomitant left-sided valve surgery. Current ESC and American College of Cardiology (ACC)/American Heart Association (AHA) guidelines support this approach (Class I), and also recommend tricuspid valve (TV) repair in moderate TR with annular dilation (Class IIa) [70,71]. In this context, transcatheter tricuspid valve interventions (TTVIs) are emerging as a viable alternative for patients deemed inoperable or high-risk [72]. TTVIs encompass several approaches: edge-to-edge repair (TEER), annuloplasty, orthotopic valve replacement, and heterotopic caval valve implantation (CAVI). Among these, TEER systems such as TriClip™ and PASCAL™ are the most widely adopted. The TRILUMINATE Pivotal trial randomized 572 patients with symptomatic severe TR to TEER plus medical therapy or medical therapy alone [73]. At 24 months, TEER led to a 28% reduction in recurrent HF hospitalizations and a significantly higher freedom from death, surgery, or repeat intervention (77.6% vs. 29.3%) [74]. TR reduction to ≤moderate severity was achieved in 84% of the TEER group versus 21% in controls, with durable QoL gains as measured by Kansas City Cardiomyopathy Questionnaire (KCCQ) scores [73,74]. Annular reduction devices such as Cardioband™ offer potential benefits in patients with atrial functional TR but pose technical challenges due to the anatomy of the tricuspid annulus and its proximity to the right coronary artery [75]. Orthotopic valve replacement with dedicated devices (e.g., EVOQUE™, Lux-Valve™) is evolving rapidly, offering the potential for more definitive treatment, but is currently limited by anatomical constraints and procedural complexity [75]. CAVI with devices such as TricValve™ represents an emerging option for patients with severe symptomatic TR who are ineligible for surgery or orthotopic transcatheter repair. The TRICUS EURO study assessed TricValve™ in high-risk patients and showed favorable short-term outcomes in terms of New York Heart Association (NYHA) class improvement and congestion relief, though long-term data are still lacking [76]. While these technologies show promise, anatomical challenges and procedural complexity persist, particularly with annular and replacement systems [76]. Despite technological progress, evidence gaps remain. Most trials have prioritized surrogate endpoints (e.g., NYHA class, KCCQ) rather than hard outcomes like mortality. Importantly, data from the just published international TRIGISTRY registry provide further insights into real-world prognosis [77]. In over 2300 patients with severe isolated TR, surgical and transcatheter interventions were associated with a significant survival benefit compared to medical therapy in those with preserved LVEF, but not in patients with mildly reduced or reduced LVEF. Notably, residual TR after TTVI was linked to worse survival, underscoring its relevance as a prognostic marker across all LVEF categories. These findings highlight the need for careful patient selection and procedural success as critical determinants of benefit, particularly in individuals with impaired LV systolic function [77]. These considerations underline that careful anatomical and functional assessment is essential to guiding patient selection, while the lack of robust mortality data highlights persisting uncertainties regarding long-term outcomes. In conclusion, TR should no longer be viewed as a benign bystander in HF. It is a modifiable driver of clinical deterioration. With the advent of transcatheter therapies, a paradigm shift is underway—from late-stage palliation to early, proactive intervention. Future research must aim to solidify TTVIs within HF management algorithms, optimizing timing, selection, and long-term outcomes.

## 6. Pushing the Limits: New Frontiers in Advanced Heart Failure

‘Advanced’, ‘refractory’, and ‘end-stage’ HF are terms describing a specific subset of HF patients, which evolves towards a significant functional limitation, regardless optimal medical therapies. In this condition, the risk benefit ratio of treatments such as Heart Transplantation (HTx) or Durable MCS becomes favorable, opening the scenario for a first evaluation for eligibility [78,79]. In the following paragraph, our aim will be to summarize the most recent updates available in the field, focusing on the innovations in managing patients following HTx or MCS.

### 6.1. Donor Derived Cell-Free DNA: The New Gold Standard in Diagnosing Rejection?

Current post-transplant surveillance strategies rely on a combination of laboratory testing, echocardiography, endomyocardial biopsy (EMB), and coronary angiography. While effective, these protocols are invasive, variably sensitive, and suffer from interobserver variability. In this context, dd-cfDNA, has emerged as a promising non-invasive biomarker for early detection of cardiac allograft injury. These DNA fragments, released during donor myocyte necrosis and apoptosis, rise in the circulation in response to immune-mediated injury, offering real-time insight into graft health [79]. Observational studies have yielded encouraging results, indicating that dd-cfDNA could help identify high-risk patients in whom the benefits of invasive procedures may outweigh the risks [80]. For instance, a prospective study investigating the correlation between dd-cfDNA and cardiac allograft rejection demonstrated that this biomarker can effectively differentiate between healthy grafts and those at elevated risk of complications in both adult and pediatric recipients. Notably, dd-cfDNA was able to discriminate among grafts experiencing acute cellular rejection (ACR), antibody-mediated rejection (AMR), and cardiac allograft vasculopathy, with a reported negative predictive value (NPV) of 80% [10]. Another study reported that, at 28 days post-HTx, an increase in dd-cfDNA above 0.25% had a sensitivity of 81%, a specificity of 85%, and an impressive NPV of 99.2% for detecting acute rejection. This approach could potentially eliminate the need for up to 80% of EMBs, while also offering the ability to distinguish between ACR and AMR. For example, dd-cfDNA levels tend to be higher and rise earlier in AMR, with distinct differences in DNA fragment length and composition. The high specificity of dd-cfDNA has led researchers to suggest that, even in cases with negative EMB findings, elevated dd-cfDNA levels may warrant closer clinical surveillance—particularly relevant given that EMB may occasionally miss subclinical or “silent” rejection. Thus, dd-cfDNA holds potential as a future gold standard in rejection monitoring [81]. These findings suggest that cf-DNA could reduce reliance on routine biopsies, facilitating earlier and more precise risk stratification. However, unresolved questions persist regarding its role in guiding therapy, tracking treatment response, and identifying subclinical pathology. Prospective interventional studies are essential before routine integration into clinical algorithms [82].

### 6.2. Donation After Circulatory Death in Heart Transplantation: Unlocking New Donor Potential

Limited organ availability remains the primary barrier to access HTx. Despite expanded acceptance of marginal donors, the donor-to-recipient ratio has steadily declined, resulting in prolonged waitlist times and increased mortality among transplant candidates [83]. Traditionally, orthotopic HTx has relied on donors after brain death (DBD), wherein organ procurement is initiated following confirmation of irreversible cessation of all cerebral activity, using cold preservation techniques under controlled conditions. DCD represents a promising alternative pathway. In this approach, patients with irreversible neurologic injury who do not meet criteria for brain death undergo withdrawal of life-sustaining therapy, and death is declared based on circulatory criteria. Two primary procurement strategies are employed: (1) direct procurement with ex vivo reperfusion using an organ care system, and (2) regional normothermic in situ perfusion with extracorporeal circulation to maintain physiological organ viability [11]. The clinical viability of DCD HTx was recently validated in a randomized, unblinded trial demonstrating non-inferiority compared with the conventional DBD approach [84]. This finding represents a significant milestone in the evolution of HTx. However, important challenges remain. The implementation of a DCD program requires substantial institutional infrastructure, interdisciplinary coordination, and ethical oversight. Clinical implications are also considerable: for instance, the duration of warm ischemia—defined as the interval between circulatory arrest and initiation of organ perfusion—varies widely by jurisdiction, from 2 to 30 min, and has a direct effect on graft function and survival [83]. Despite the overall favorable findings, the trial revealed that 15% of recipients of DCD hearts experienced severe primary graft dysfunction within 30 days, compared to only 5% among recipients of DBD organs. Although most dysfunctions were manageable, two patients required retransplantation. Warm ischemia is believed to be a primary contributor to this difference and warrants further investigation [84]. Another limitation of the trial was its unblinded design, which introduced potential selection bias. Notably, a larger proportion of patients with higher United Network for Organ Sharing (UNOS) status (1 and 2) received DBD organs, possibly influencing outcome comparisons [82]. Despite these limitations, DCD transplantation offers a viable strategy to expand the donor pool and reduce waitlist mortality. As clinical protocols are optimized and long-term data accumulate, DCD is likely to play an increasingly central role in addressing the donor organ shortage. A comparative overview of HTx from circulatory death versus DBD is summarized in Table 4.

### 6.3. Update in Management of LVAD Patients

LVAD represent a pivotal therapeutic option for patients with advanced HF who are either ineligible for HTx or are experiencing progressive clinical deterioration while awaiting a donor organ. Recent advancements in the management of these patients have markedly improved long-term outcomes and QoL by enhancing device durability and minimizing hemocompatibility-related adverse events (HRAEs). The evolution from large, pulsatile devices to compact, intrapericardial, continuous-flow systems has led to a reduction in complications and improved survival. Minimally invasive surgical approaches, including thoracotomy and upper hemisternotomy, are increasingly utilized in experienced centers to further reduce perioperative morbidity. Among currently available systems, the HeartMate 3 device (Abbott, Chicago, IL, USA), a fully magnetically levitated centrifugal pump, has demonstrated superior outcomes, with significantly lower rates of pump thrombosis and disabling strokes, while maintaining similar risks of bleeding and driveline infections [85,86]. Technological innovations continue to address persistent limitations, particularly driveline-related complications. Wireless power transfer systems are under development, although engineering challenges remain, including optimizing energy efficiency, thermal management, miniaturization, and long-term biocompatibility. Early prototypes and pilot studies have yielded encouraging results [87]. Current studies also provided insights into the medical management of LVAD recipients. Anticoagulation protocols are evolving to reflect improved hemocompatibility of newer devices and deeper understanding of bleeding and thrombosis risks [85]. As current guidelines recommend, LVAD patients are treated with vitamin K antagonists (e.g., warfarin) and antiplatelet agents (usually low-dose aspirin). A more personalized, risk-adjusted approach is emerging, prioritizing safety from bleeding complications while maintaining effective thromboembolic protection. The ARIES-HM3 study showed that omission of aspirin in HeartMate 3 LVAD recipients is associated with a significant reduction in non-surgical bleeding, particularly gastrointestinal bleeding, without increasing the risk of major HRAEs [87]. A secondary analysis confirmed that this aspirin-free regimen is safe even in presence of a prior atherosclerotic vascular condition (prior percutaneous coronary intervention, coronary artery bypass grafting, stroke, or peripheral vascular disease) [88]. Thus, the use of aspirin in this setting will likely decrease significantly. There is also growing interest in the use of DOACs as alternatives to warfarin. Early data from small studies and case series suggest that DOACs might be safe and effective in patients with newer-generation devices. The DOAC LVAD study revealed that therapy with apixaban administered twice daily at a 5 mg dose was not associated with an increase in death or HRAEs, with the predominant effect to reduce the risk of major bleeding compared with warfarin therapy [89]. Enrolled patients had a HeartMate 3 device and received antiplatelet therapy with low-dose aspirin throughout the trial. The DOT HeartMate 3 study assessed the feasibility of using apixaban without aspirin in carefully selected stable patients even if implanted as bridge to transplant strategy [12]. Although this promising result, large, randomized trials are still needed before widespread adoption.

### 6.4. New Generation Total Artificial Heart: From Vision to Viable Lifeline

The Total Artificial Heart (TAH) is a form of MCS in which the patient’s native ventricles and valves are completely removed and replaced. TAH offers a potential solution as a bridge to HTx for patients with end-stage biventricular HF who are not eligible for LVAD implantation, particularly in countries where donor organ availability is limited. High complication rates have limited TAH widespread adoption in the past. The development of the Aeson TAH (CARMAT), however, has renewed interest in this therapy. This new-generation device incorporates several innovative features that aim to closely mimic natural cardiac physiology: biocompatibility by using bovine pericardium in blood-contacting surfaces, pulsatile blood flow generated by hydraulic pumps imitating natural systolic and diastolic phases and real-time autoregulation of output based on the patient’s physiological needs while also preventing stroke and bleeding complications [90,91]. A small study demonstrated that Aeson TAH can be effectively used as a bridge to HTx in patients with severe pulmonary hypertension allowing for continuous hemodynamic monitoring and support in these high-risk transplant candidates [13]. Ongoing clinical trials are essential to further assess its long-term safety and efficacy and its potential to become a destination therapy. EFICAS trial (ClinicalTrials.gov ID NCT04475393) has recently concluded enrollment to demonstrate the safety and efficacy of Aeson TAH as a bridge to HTx, focusing on stroke-free survival at 6 months [92,93]. Results are expected by the end of 2025. The integration of advanced materials and technologies positions the Aeson TAH as a potential game-changer in the field of MCS.

## 7. Conclusions and Future Directions

HF management is entering a new era—one defined by earlier intervention, multidimensional care, and expanding therapeutic horizons. While the foundational “four pillars” of therapy have revolutionized outcomes in HFrEF, their full potential remains constrained by suboptimal implementation in real-world practice. Novel agents like finerenone are reshaping the cardio–renal–metabolic interface, offering targeted protection for patients with T2DM and CKD, while GLP-1 RAs emerge as intriguing candidates for CV risk reduction, particularly in patients with HFpEF and obesity. Likewise, TR has evolved from a neglected marker to a therapeutic priority, with transcatheter therapies redefining the management landscape. Yet, the most formidable frontier remains advanced HF—a domain where prognosis is poor, and access to HTx continues to decline. In this context, innovations such as DCD and total artificial hearts represent critical avenues for expanding treatment options. However, both approaches should still be regarded as emerging strategies, with limited long-term outcome data and considerable challenges for widespread implementation. The most recent evidence underscores how the therapeutic armamentarium continues to evolve. The DIGIT-HF (Digitoxin in Patients with Heart Failure and Reduced Ejection Fraction) trial demonstrated that the addition of digitoxin to standard therapy in patients with HFrEF significantly reduced the composite endpoint of all-cause death or HF hospitalization compared with placebo (HR 0.86; 95% CI 0.78–0.95) [94]. Similarly, the VICTOR trial evaluating vericiguat showed that, although the primary composite endpoint was not met, a reduction in CV mortality was observed, suggesting a potential role in carefully selected high-risk subgroups [95]. The path forward demands an integrated approach—one that combines optimized GDMT, incorporation of novel agents, cutting-edge device-based interventions, and system-level strategies to ensure equitable access and individualized care across the HF spectrum. Precision-guided, multidisciplinary management represents the cornerstone of this new era, offering the best opportunity to improve prognosis and reduce the global burden of HF.

## Figures and Tables

**Table 1:** Real-world adoption and optimization of guideline-directed medical therapy in heart failure with reduced ejection fraction.

Trial (Year)	Design	n/Population	Control Group/Setting	Main Findings
EVOLUTION-HF (2024) [15]	Retrospective analysis (real-world, multi-country)	~600,000 HF patients across multiple countries	Observational (secondary data)	Delayed initiation of dapagliflozin and sacubitril/valsartan; frequent discontinuation of ACEis/ARB/BB/MRA; higher persistence with SGLT2is
GWTG-HF (USA, >100k) [16]	National registry, quality improvement program (non-randomized)	Hospitalized HF patients	Registry-based comparison	Low post-discharge initiation of SGLT2is (<20%) in eligible patients; suboptimal GDMT uptitration
TITRATE-HF (NL, 2023) [17]	Prospective national registry	4288 patients with de novo, chronic, or worsening HFrEF	Routine clinical practice	Quadruple therapy in 44% of chronic/worsening HFrEF; only 1% reached target doses; specialized HF clinics improved implementation
BRING-UP-3 HF (Italy, 2024) [18]	National implementation science initiative	3830 ambulatory HF patients (58.4% HFrEF)	Real-world, multicenter program	Quadruple therapy in 65% of HFrEF; >90% BBs and RASi/ARNI use; >80% SGLT2is and MRA use; SGLT2is uptake also high in HFmrEF (72%) and HFpEF (50%)

Abbreviations: ACEis = angiotensin-converting enzyme inhibitors; ARB = angiotensin receptor blocker; BBs = beta-blockers; GDMT = guideline-directed medical therapy; HFrEF = HF with reduced EF; HFmrEF = HF with mildly reduced EF; HFpEF = HF with preserved EF; MRA = mineralocorticoid receptor antagonist; RASi = renin–angiotensin system inhibitor; SGLT2is = sodium–glucose cotransporter-2 inhibitors.

**Table 2:** Clinical development of finerenone across the HF–CKD–T2DM spectrum.

Trial (Year)	Design	n	Inclusion Criteria	Control Group	Primary Endpoint	Main Findings
ARTS (2013) [33]	Double-blinded	457	HFrEF (≤40%) and mild-moderate CKD	Placebo and Spironolactone	BNP, amino-terminal proBNP, albuminuria, hyperkalemia	Similar natriuretic peptide and albuminuria reduction Lower incidence of hyperkalemia (5.3% vs. 12.7%, *p* = 0.048)
ARTS-HF (2016) [34]	Double-blinded	1066	HFrEF and CKD and/or T2DM	Eplerenone	% of patients with a decrease of >30% in NT-proBNP	Similar NT-proBNP reduction at different Finerenone dosages
FIDELIO-DKD (2020) [35]	Double-blinded	5734	CKD and T2DM (~8% with HF)	Placebo	Composite of AKI, CKD progression and death from renal causes	Lower risk of composite outcome (HR 0.82, 95% CI 0.73–0.93)
FIGARO-DKD (2021) [36]	Double-blinded	7437	CKD and T2DM (~8% with HF)	Placebo	Composite of CV death, MI, stroke, HF hospitalization	Lower risk of composite outcome (HR 0.87, 95% CI 0.76–0.98) Benefit driven primarily by a lower incidence of hospitalization for HF (HR 0.71, 95% CI 0.56–0.90)
FIDELITY (2022) [37]	Pooled analysis	13,026 (pooled)	CKD and T2DM (~8% with HF)	Placebo	Composite CV outcome and composite kidney outcome	Lower risk of composite CV outcome (HR 0.86, 95% CI 0.78–0.95) Lower risk of composite kidney outcome (HR 0.77, 95% CI 0.67–0.88)
FINEHEART-HF (2024) [38]	Double-blinded	6001	HFmrEF and HFpEF (LVEF ≥40%)	Placebo	Composite of worsening HF or CV death	Lower risk of composite outcome (HR 0.84, 95% CI 0.74–0.95) Lower risk of worsening HF (HR 0.82, 95% CI 0.71–0.94) Similar CV death (HR 0.93, 95% CI 0.78–1.1)

Abbreviations: AKI = acute kindney injury; BNP = B-type natriuretic peptide; CI = confidence interval; CKD = chronic kidney disease; CV = cardiovascular; HF = heart failure; HFmrEF = HF with mildly reduced ejection fraction; HFpEF = HF with preserved ejection fraction; HFrEF = HF with reduced ejection fraction; HR = Hazard ratio; LVEF = left ventricular ejection fraction; NT-proBNP = N-terminal pro-B-type natriuretic peptide; T2DM = type 2 diabetes mellitus.

**Table 3:** Key clinical studies of GLP-1 receptor agonists in heart failure.

Trial (Year)	Design	n	Inclusion Criteria	Control Group	Primary Endpoint	Main Findings
AMPLITUDE-O (2021) [55]	Double-blinded	4076	T2D and either a history of CV disease or CKD (≈18% history of HF)	Placebo	First MACE (CV death, myocardial infarction, or stroke)	Risk of outcome was lower among those who received weekly subcutaneous injections of Efpeglenatide at a dose of 4 or 6 mg (HR 0.73, 95% CI 0.58–0.92)
Harmony Outcomes (2018) [56]	Double-blinded	9463	T2D and either a history of CV disease or CKD (≈20% history of HF)	Placebo	First MACE (CV death, myocardial infarction, or stroke)	Albiglutide was superior to placebo in lowering the primary outcome (HR 0.78, 95% CI 0.68–0.90)
EXSCEL (2017) [59]	Double-blinded	14,752	T2D, with or without history of CV disease (≈19% history of HF)	Placebo	First MACE (CV death, myocardial infarction, or stroke)	Subcutaneous injections of extended-release Exenatide at a dose of 2 mg once weekly not superior to placebo with respect to efficacy (HR 0.91, 95% CI 0.83–1.00)
LIVE (2017) [60]	Double-blinded	241	HF (LVEF ≤45%), with or without history T2D	Placebo	Change in LVEF	Liraglutide 1.8 mg once daily did not affect left ventricular systolic function

Abbreviations: CI = Confidence interval; CKD = chronic kidney disease; CV = Cardiovascular; HF = Heart failure; HR = Hazard ratio; LVEF = Left ventricular ejection fraction; MACE = Major adverse cardiovascular events; T2DM = Type 2 diabetes mellitus.

**Table 4:** Comparative overview of donation after brain death versus donation after circulatory death.

Domain	Donation After Brain Death	Donation After Circulatory Death
**Definition**	Organ procurement following confirmed irreversible cessation of all brain function	Organ procurement after cessation of circulatory and respiratory activity post withdrawal of support
**Donor Selection Criteria**	Neurologically deceased individuals meeting legal and clinical brain death criteria	Patients with irreversible brain injury but not fulfilling brain death criteria
**Hemodynamic Stability**	Maintained via mechanical ventilation and vasopressors	Rapid deterioration post support withdrawal; variable ischemic time
**Organ** **Preservation**	Immediate cold perfusion under controlled conditions	Ex vivo (e.g., Organ Care System) or regional in situ normothermic perfusion
**Warm Ischemia Time**	Negligible	Variable (2–30 min); critical for graft function
**Graft Function**	Low rates of primary graft dysfunction (PGD)	Slightly higher PGD; improving with technique optimization
**Infrastructure**	Standard; well-established across centers	Requires specialized infrastructure and rapid-response logistics
**Ethical** **Considerations**	Clear protocols and legal frameworks	Requires societal trust, transparent consent, and ethical oversight
**Clinical** **Outcomes**	Excellent short- and long-term outcomes	Non-inferior to DBD in selected populations; long-term data still accruing
**Advantages**	Predictable logistics; high success rate	Expands donor pool; reduces waiting list times
**Limitations**	Limited donor availability tied to brain death	Warm ischemia risk; resource-intensive implementation

Abbreviations: DBD = Donation After Brain Death; DCD = Donation After Circulatory Death; PGD = Primary Graft Dysfunction; OCS = Organ Care System.
